# Direct Comb Vernier Spectroscopy for Fractional Isotopic Ratio Determinations

**DOI:** 10.3390/s21175883

**Published:** 2021-08-31

**Authors:** Mario Siciliani de Cumis, Roberto Eramo, Jie Jiang, Martin E. Fermann, Pablo Cancio Pastor

**Affiliations:** 1 Agenzia Spaziale Italiana, Contrada Terlecchia SNC, 75100 Matera, Italy; 2 Istituto Nazionale di Ottica, INO-CNR, Via N. Carrara 1, 50019 Sesto Fiorentino, Italy; roberto.eramo@ino.cnr.it (R.E.); pablo.canciopastor@ino.cnr.it (P.C.P.); 3 Dipartimento di Fisica, Universitá degli Studi di Firenze, Via G. Sansone 1, 50019 Sesto Fiorentino, Italy; 4 IMRA America, Inc., 1044 Woodridge Avenue, Ann Arbor, MI 48105, USA; jjiang@imra.com (J.J.); mfermann@imra.com (M.E.F.)

**Keywords:** isotopic ratio, frequency comb, Vernier spectroscopy

## Abstract

Accurate isotopic composition analysis of the greenhouse-gasses emitted in the atmosphere is an important step to mitigate global climate warnings. Optical frequency comb–based spectroscopic techniques have shown ideal performance to accomplish the simultaneous monitoring of the different isotope substituted species of such gases. The capabilities of one such technique, namely, direct comb Vernier spectroscopy, to determine the fractional isotopic ratio composition are discussed. This technique combines interferometric filtering of the comb source in a Fabry–Perot that contains the sample gas, with a high resolution dispersion spectrometer to resolve the spectral content of each interacting frequency inside of the Fabry–Perot. Following this methodology, simultaneous spectra of ro-vibrational transitions of ${}^{12}$C${}^{16}$O${}_{2}$ and ${}^{13}$C${}^{16}$O${}_{2}$ molecules are recorded and analyzed with an accurate fitting procedure. Fractional isotopic ratio ${}^{13}$C/${}^{12}$C at 3% of precision is measured for a sample of CO${}_{2}$ gas, showing the potentialities of the technique for all isotopic-related applications of this important pollutant.

## 1. Introduction

Measuring the isotope ratio of chemical substances (carbon, water, chlorine and so on) has a large variety of applications in environmental sciences [1,2,3,4,5]. Providing a monitor of the emission in the atmosphere of greenhouse gases has potential application in global climate warnings, as well as in general monitoring [6,7,8,9], water cycle studies [10,11,12], and in general for establishing formation mechanisms and strategies [13,14,15,16,17]. Additionally, biomedical applications benefit from accurate fractional isotopic ratio measurements, particularly in human breath analysis [18,19], where it is possible to detect biomarkers related to specific diseases or metabolic processes, or even in pharmacological research [20]. Finally, isotopic ratio measurements are employed in space research, in star dynamics and planet studies [21]; the particular measurement of the ${}^{13}C{/}^{12}C$ ratio, which we consider in this work, has additional interests in carbon capture and storage monitoring [22] and in volcanic gas processes studies [23]. According to the type of application, the desirable target for accuracy and precision in such measurements could be different. For example, in very demanding biomedical applications (i.e., breath test for disease diagnosis or metabolic status monitoring), the accuracy and precision level for the $R_{{}^{13}C{/}^{12}C}$ isotopic ratio could be lower than 0.5% [24,25]

Broadband spectroscopic techniques that use optical frequency combs (OFC) as an interaction laser source have become very popular for multiplexed detection of molecular species. Among all molecular parameters measurable with such techniques, the accurate determination of the isotopic composition of a gas sample is surely one of the most challenging applications.

OFC-based spectroscopic techniques with dual comb [26], Fourier transform [27] and spatially dispersive [28,29,30,31] detection schemes, sometimes combined with Fabry–Perot enhanced spectroscopy [29,30,31,32], are widely used for trace gas detection [19,30,31,33,34,35,36,37]. However, the most accurate results regarding fractional isotopic ratio measurements were obtained by direct frequency comb dispersive spectroscopy (DFCDS) [25,38]. Bailey and coworkers [38] used a MIR-FC-based cross-dispersed spectrometer to measure a fractional isotopic abundance of nitrous oxide (N${}_{2}$O) with a precision of 6.7 × 10${}^{-6}$ in 1 s of acquisition time. Such measurements can be used to determine sources, skins and mechanisms of formation of this potent greenhouse gas and ozone-depleting agent, helping to improve current mitigation strategies. Similarly, accurate optical number density of ${}^{12}C$ and ${}^{13}C$ single substituted isotopic variants of C${}^{16}$O${}_{2}$ gas with a precision of, respectively, 0.03% and 1.24%, were measured by using DFCDS in the near-IR [25]. These kinds of measurements open the way for environmental monitoring and biomedical sciences applications of this OFC-based technology. DFCDS exploits the broadband coverage and spectroscopic resolution and irradiance of the laser source to allow simultaneous detection of almost all isotopic components of the targeted gas with short acquisition times and with a compact technology. The accuracy of the resulting fractional isotopic ratio measurements are comparable to single-frequency-laser-based spectrometers [7,8,18,39,40,41,42] and to the mass spectrometry performance currently used for these kinds of measurements. Such accuracy is ensured by a not-trivial calibration of the spectral instrumental response of the DFCDS apparatus in order to identify the contribution of each interacting frequency of the comb with the sample gas. In this article, we report the capabilities of a slightly modified DFCDS approach, called direct comb Vernier spectroscopy (DCVS) [29,35], to perform fractional ratio isotopic measurements in ${}^{12}$C${}^{16}$O${}_{2}$ and ${}^{13}$C${}^{16}$O${}_{2}$ components of a CO${}_{2}$ gas sample around 5005 cm${}^{-1}$.

Our DCVS combines efficient comb filtering by using interferometric Fabry–Perot (FP) techniques in an adequate Vernier configuration with the spectral resolution of the FP-transmitted comb by using a high-resolution dispersive spectrometer. In addition, our spectrometer well isolates each of the sample-interacting teeth of the OFC from the others, and their spectral contribution can be extracted by using a simpler instrumental response approach, opening the way to very accurate lineshape studies. As a drawback, the detected OFC portions are quite limited compared to DFCDS, reducing the possibilities to reach a larger number of isotopologues of the gas in a single acquisition. Nevertheless, the reported fractional ${}^{13}$C/${}^{12}$C at 3% of precision is a proof of the principle of the capabilities of this technique for these kinds of measurements.

## 2. Materials and Methods

The DCVS apparatus used for the present measurements is described in detail elsewhere [29,35]. Basically, an OFC is spectrally filtered by means of a Fabry–Perot (FP) cavity acting as an interaction cell containing the absorbing gas. The Vernier ratio (*V*) between the FP and the OFC (i.e., the ratio between the FP’s free spectral range $\Delta_{FSR}$ and the OFC’s repetition rate, $f_{r}$) is established to be enough to resolve the FP-transmitted OFC modes with a high-resolution dispersion spectrometer (SOPRA, resolution 2 GHz @ 2 μm). Indeed, after FP-filtering, spectral fractions of the OFC of about 5 cm${}^{-1}$ are dispersed at the output of the SOPRA instrument and detected with an InGaAs linear array detector. Different from the experimental setup described in [29,35], the OFC is a thulium-doped fiber-based mode-locked laser from IMRA America, Inc. It has a spectral coverage of about 40 nm to around 1970 nm with a repetition rate of about 400 MHz, with a maximum averaged power of about 4 W after the final amplification stage. The OFC is a self-referenced system, employing the first amplification stage (about 1 W of avg. power) to generate OFC emission around 1 μm through to the non-linear optical processes in optical fibers. The carrier-offset-frequency parameter of the OFC, $f_{o}$, is consequently detected by beating the teeth frequencies of the duplicated fraction of the fundamental comb emission with those of the 1 μm harmonic, and controlled by means of the phase-lock-loop (PLL) against the stable RF clock. The repetition frequency, $f_{r}$, is controlled by detecting the beat of the comb modes with a fast InSb photodetector and by mixing it with RF synthesized frequency ($f_{RF}$) to obtain the RF note at 150 MHz locked with a second PLL circuit. The reference clock for both $f_{r}$ and $f_{o}$ locks is a 10 MHz quartz-Rb-GPS system with a relative frequency stability of 6 × 10${}^{-13}$ in 1 s and an accuracy of 10${}^{-12}$, at worst. Hence, the frequency of the OFC modes is directly traceable to the primary frequency standard with these precision and accuracy figures when locked.

**Figure 1:**
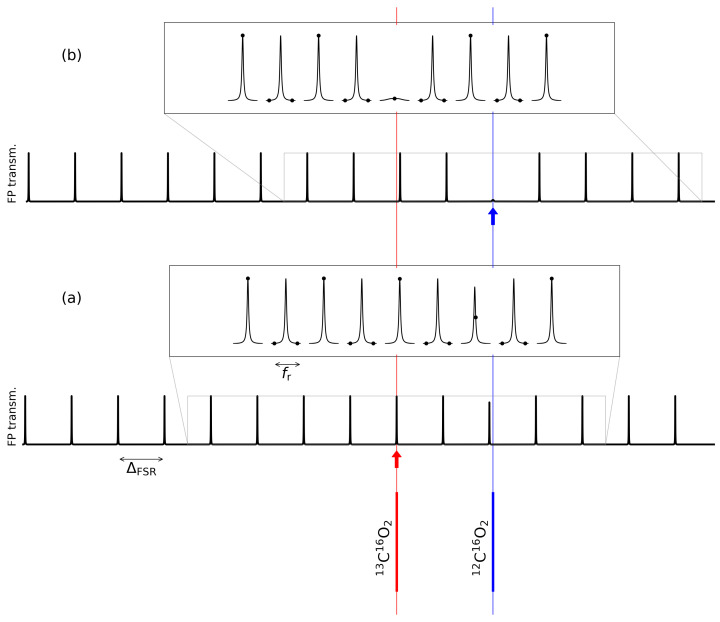
The two roto-vibrational transitions are probed around the two comb/FP configurations shown in figure, where the FP transmission as a function of frequency is shown in two cases. In case (**a**), one comb tooth and one FP mode are resonant with the ${}^{13}$C${}^{16}$O${}_{2}$ transition, while in case (**b**), they are resonant with the ${}^{12}$C${}^{16}$O${}_{2}$ transition. Far from resonances, the FP mode is an Airy function; around a resonance frequency, the mode is modified by the resonant refractive index of the FP medium. Here, the simulation is made by taking Voigt profiles for the two investigated transitions (Hitran parameters), and with f${}_{r}$ = 400 MHz, $\Delta_{FSR}=6.2$ GHz (i.e., $2\Delta_{FSR}/f_{r}=31$), and a FP finesse F=200. In each inset immediately above the FP transmission, each FP mode is zoomed (broken axis plot). We observe that one FP mode every two transmits a comb tooth, and that between adjacent zoomed FP modes, there are 15 comb modes not transmitted and not shown in the inset. We observe that in the (**a**) case, both transitions are simultaneously probed by different comb teeth, the ${}^{13}$C${}^{16}$O${}_{2}$ transition giving an effect not visible in the graph. The (**b**) case shows a heavily saturated ${}^{12}$C${}^{16}$O${}_{2}$ transition.

The FP is a linear cavity with silver-coated mirrors. The finesse is about *F* = 200 @ 2 μm with a transmission coefficient around 0.2%. The cavity length is variable and coarsely controllable by means of step-motors mounted in the kinematic mount of one of the two cavity mirrors. It’s value is established by the chosen Vernier ratio *V* and by the requirement to be long enough to obtain an efficient enhancement of the absorption path. For the present measurements, where a simultaneous detection of the comb modes resonant with transitions of different isotopologues of the targeted molecule is needed, *V* should be fixed to a value which gives a $\Delta_{FSR}$ as close as possible to a multiple of the isotope shift between the probed molecular transitions. Transitions of the ${}^{12}$C and ${}^{13}$C isotopes of the carbon dioxide molecule around 5005 cm${}^{-1}$ are used to test the spectroscopic performances of the technique. In particular, the (20${}^{0}$1–00${}^{0}$0) R(18) of ${}^{13}$C${}^{16}$O${}_{2}$ @ 5004.84 cm${}^{-1}$ and the (20${}^{0}$1–00${}^{0}$0) P(45) of ${}^{12}$C${}^{16}$O${}_{2}$ @ 5005.27 cm${}^{-1}$ ro-vibrational transitions are selected in order to match the condition of simultaneous recording of both absorptions in the detected range of 5 cm${}^{-1}$ of the cavity-transmitted and dispersed fraction of the interacting OFC for our spectrometer. The frequency shift between these two transitions is around $\Delta \nu^{IS}$ = 12,863.55 MHz. A $\Delta_{FSR}$ of the order of half of $\Delta \nu^{IS}$ allows one to obtain an enhanced absorption path of about 2 m (i.e., $L_{FP}$ = 2.4 cm), while keeping the FP mode separation more than three times larger than the SOPRA spectral resolution. In addition, a non-integer *V* ratio is chosen to obtain rarefied resonance between OFC and FP while keeping the condition that the two FP transmissions are in resonance with the two selected CO${}_{2}$ transitions. Indeed, choosing *V* = 15.5 (i.e., 31 comb modes each 2 FP modes) tailors this condition, further helping in better FP-filtering of the OFC and a better resolved image of the transmitted modes by the SOPRA diffraction spectrometer. In Figure 1 is shown a scheme of the measurements: in one case the comb/FP configuration shown is resonant with the center of ${}^{13}$C${}^{16}$O${}_{2}$ transition. In such a case as shown in Figure 1a, both transitions are simultaneously probed by different comb teeth, while in Figure 1b, only the ${}^{12}$C${}^{16}$O${}_{2}$ transition shows an heavily saturated absorption.

The DCVS is performed under the condition of perfect resonance between the FP and the transmitted OFC modes for each value of their optical frequency. Consequently, the FP length is actively locked to set it on resonance with the maximum transmission of the Vernier resonant modes. To this aim, one of the FP mirrors is mounted in a PZT to obtain fine control of the cavity length by detecting a small part of the FP-transmitted light in a InGaAS detector. A 3 kHz modulation is applied to the PZT, and the first derivative of the FP output detected light is obtained by means of locking detection and used as an error signal to control the cavity length to the maximum transmission condition.

**Figure 2:**
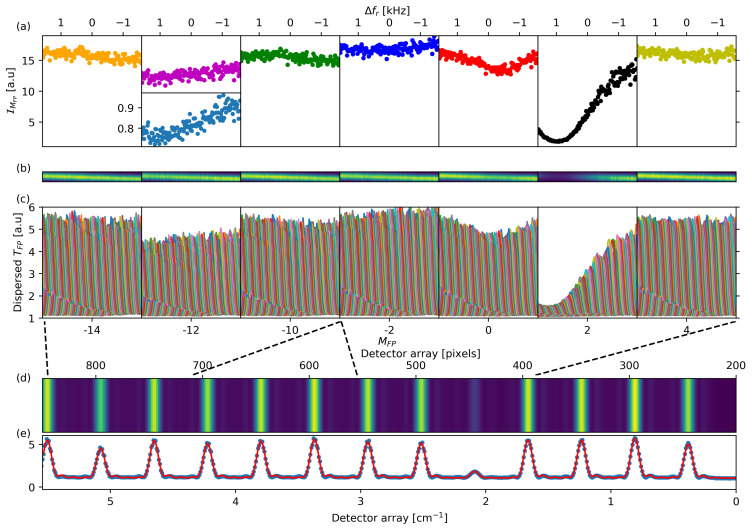
Spectra of the OFC modes filtered by the FP as a function of the OFC’s $\Delta f_{r}=f_{r}-f_{r_{c}}$, with $f_{r_{c}}$ repetition rate frequency at the center of the scan. The spectra are the result of the analysis of the recorded images of the SOPRA-dispersed fraction of the FP-filtered OFC vs $f_{r}$. Three of these modes are resonant with the (20${}^{0}$1-00${}^{0}$0) R(14) of ${}^{13}$C${}^{16}$O${}_{2}$ @ 5002.21 cm${}^{-1}$, (20${}^{0}$1-00${}^{0}$0) R(18) of ${}^{13}$C${}^{16}$O${}_{2}$ @ 5004.84 cm${}^{-1}$, and (20${}^{0}$1-00${}^{0}$0) P(45) of ${}^{12}$C${}^{16}$O${}_{2}$ @ 5005.27 cm${}^{-1}$ ro-vibrational transitions; the other modes are not absorbed by the CO${}_{2}$ molecule. The modes are identified by the FP order scale of panel (**c**): the −12, 0 and +2 orders are the modes absorbed by ${}^{13}$C${}^{16}$O${}_{2}$ and ${}^{12}$C${}^{16}$O${}_{2}$ transitions, respectively; the −14, −10, −2 and +4 orders are the not-absorbed ones. (**a**) Integrated transmission spectrum. (**b**) Spectral behavior of the array images for each mode. (**c**) Spectral behavior of the SOPRA-resolution wavelet for each mode. (**d**) Detector array image of the portion of the FP-transmitted OFC modes for a given $f_{r}$. (**e**) Vertically integrated intensity of the transmitted modes of image in panel (**d**) and the fit to a function resulting form the addition of 13 SOPRA-resolution functions; the intensity of the fitted resolution provides the transmission spectra values for each $f_{r}$ of panel (**a**). The inside graph of panel (**a**) shows the transmittance spectrum of the R(14) transition of the ${}^{13}$C${}^{16}$O${}_{2}$ from the M=−12 mode normalized by the average intensity of the −14 and −10 modes.

The majority of the FP-transmitted light is sent to the SOPRA input slit, and the diffracted light at the output slit, for a given position of the diffraction grating, is detected by a liquid N${}_{2}$-cooled InGaAs array detector (PyloN-IR:1024, Princeton Instr.). The arrangement of the optical components before and after SOPRA is devised to obtain the largest spectral fraction in a single array’s image, while keeping the maximum SOPRA resolution. In Figure 2d, an example of such an image is shown for $f_{r}$ = 398.99 MHz. For each image, 13 transmitted modes are detected, which is a spectral portion of the OFC of about 5 cm${}^{-1}$ taking into account the 31 comb modes for each transmitted interval. The vertically integrated intensity profile of the dispersed image (Figure 2e) is used to calculate the transmitted contribution of each detected mode. As thoroughly described in [29], a knowledge of the FP-SOPRA system resolution function, i.e., its response to a monochromatic input, is necessary in order to obtain successively the OFC modes transmissions. In this paper, in order to work with an analytical resolution function, we adopt a kind of continuous wavelets [43] approximation variant of the approach followed in [29]: we fit a single mode diode laser response by a superposition of Gaussian functions, treating their centers and widths as free parameters. In practice, a superposition made of seven of such wavelets is sufficient to reproduce the experimental diode laser response within the measurement noise. Once the resolution is determined in this way, the data analysis proceeds as in [29]: the resolution function, which is now an analytical function, is replicated on the set of N=13 transmitted OFC modes, giving a total fitting function for the integrated image with free parameters given by the position of one of the peaks, the peak’s separation, and by the *N* peaks intensities, which we write as $I_{M}\left(f_{r},P,T\right)$, where the arguments $f_{r}$, and the pressure *P* and temperature *T* of the gas sample identify the experimental conditions, and where the subscript *M* identifies each one of the N recorded FP modes per image. Figure 2e shows an example of the fit.

The absorption spectrum of the tested molecular transitions is determined by recording the set of array images for a scan of the OFC frequencies around each transition frequency. The synthesized scan of $f_{r}$ is accomplished with a change of the $f_{RF}$ frequency in the $f_{r}$-lock chain. Custom software was used to obtain an automated acquisition of such spectral images as a function of $f_{r}$. In Figure 2b, the behaviors of the detected images as a function of the $\Delta f_{r}$ for the FP-transmitted orders involved in the determination of the spectra of the ${}^{12}$CO${}_{2}$ and ${}^{13}$CO${}_{2}$ transitions are shown. For the given grating position, we label the relevant FP orders as the M=0 order, resonant with the (20${}^{0}$1-00${}^{0}$0) R(18) of ${}^{13}$C${}^{16}$O${}_{2}$ transition, the M=+2 order, resonant with the (20${}^{0}$1-00${}^{0}$0) P(45) of ${}^{12}$C${}^{16}$O${}_{2}$ transition, and the M=−2 and M=+4 orders, used to calculate the transmittance spectra not-resonant with the CO${}_{2}$ transitions. In addition, the scan behaviors for the M=−12 order, partially resonant with the (20${}^{0}$1–00${}^{0}$0) R(14) transition of ${}^{13}$C${}^{16}$O${}_{2}$, as well as those of the not CO${}_{2}$ resonant M=−14 and M=−10 modes, are shown. Figure 2c shows details of the scan of these modes as a function of the transmitted comb mode frequency, while the corresponding integrated intensities $I_{M}$ are shown in Figure 2a.

**Figure 3:**
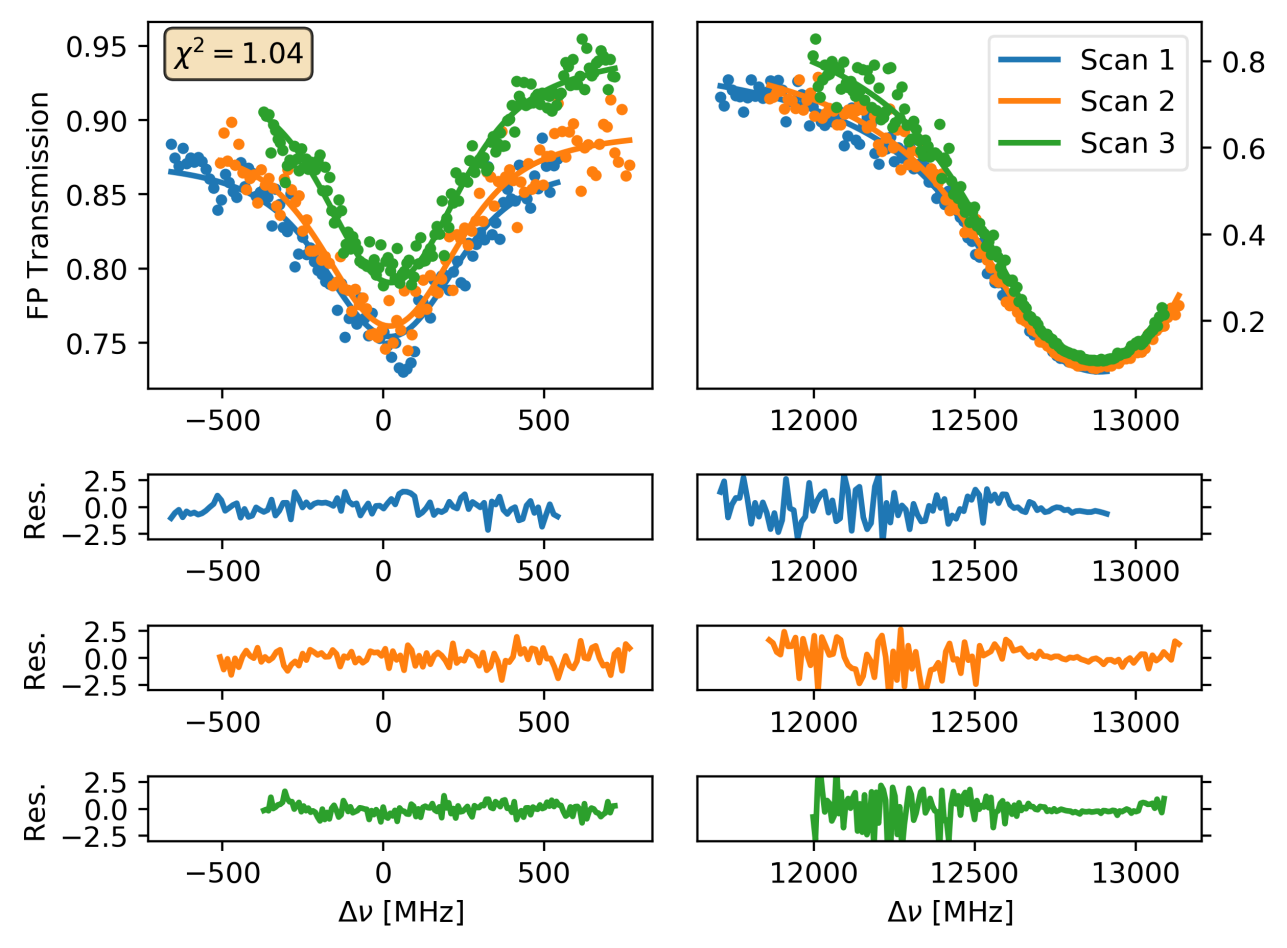
FP transmittance of the absorbed comb modes resonant with the(20${}^{0}$1-00${}^{0}$0) R(18) of ${}^{13}$C${}^{16}$O${}_{2}$ (left side graphs) and (20${}^{0}$1-00${}^{0}$0) P(45) of ${}^{12}$C${}^{16}$O${}_{2}$ (right side graphs) transitions and the fit to the T(Δν,P,T) (Equation (4)) of all recorded spectra by using the global fit procedure. P = 55.0 mbar and T = 296 K for the CO${}_{2}$ gas sample for all acquisitions. The reduced $\chi^{2}$ of the global fit is also shown.

The ratio between the integrated intensity for the molecule absorbed modes (M = 0 and +2) with the averaged intensity of the not-absorbed modes (M = −2 and +4) is used to calculate the transmittance spectrum of the ${}^{13}$CO${}_{2}$ and ${}^{12}$CO${}_{2}$ transitions, respectively, as shown in Figure 3 and in the inside graph of Figure 2a:

(1) $$\begin{array}{ccc} T_{M}^{\left(s\right)}\left(\Delta \nu ,P,T\right) & = & 2\frac{I_{M}\left(f_{r},P,T\right)}{I_{-2}\left(f_{r},P,T\right)+I_{+4}\left(f_{r},P,T\right)}\;\;\left(M=0,2\right) \end{array}$$

On the right hand-side of Equation (1) we leave as arguments the parameters $f_{r}$, *P* and *T* that set the experimental conditions of the acquisition, while for the task of the successive analysis procedure where optical frequencies are required to be compared with other results, the optical detuning Δν is used instead of $f_{r}$. Δν is calculated as the optical detuning of the *M* mode with respect to the frequency of the M=0 mode at the center of the $f_{r}$ scan:

(2) $$\begin{array}{ccc} \Delta \nu & = & N_{M}f_{r}-N_{0}f_{r_{c}}\;\;\left(M=0,2\right) \end{array}$$

with the $f_{r_{c}}$ repetition rate frequency at the center of the scan and with $N_{2}=N_{0}+31$. $N_{0}$ is calculated from the integer ratio between the reported frequency [44] of the ${}^{13}$C${}^{16}$O${}_{2}$ transition and $f_{r_{c}}$.

Due to the limited continuous scan of the $f_{r}$ in the OFC lock condition, the high frequency wings of the P(45) transition of ${}^{12}$C${}^{16}$O${}_{2}$ and of the R(14) transition of ${}^{13}$C${}^{16}$O${}_{2}$ are not recorded. Such a limitation should be overcome by using a better combination of the frequency parameters of OFC and FP. Uncertainties of the measured spectral parameters for such transitions are expected to be affected by this issue, as discussed in the following. The situation is critical for the R(14) transition of ${}^{13}$C${}^{16}$O${}_{2}$ because it is recorded by less than one half, as shown in the inside graph of Figure 2a. Consequently, it is not considered in the present isotopic ratio measurements.

## 3. Results

Three DCVS spectra of the (20${}^{0}$1–00${}^{0}$0) P(45) of ${}^{12}$C${}^{16}$O${}_{2}$ and (20${}^{0}$1–00${}^{0}$0) R(18) of ${}^{13}$C${}^{16}$O${}_{2}$ transitions are recorded with CO${}_{2}$ gas at a pressure of P = 55 mbar and at room temperature (T = 296 K). The total transmittance of the two absorbed modes for each recording is given by the following:

(3) $$\begin{array}{ccc} T^{\left(s\right)}\left(\Delta \nu ,P,T\right) & = & T_{M=0}^{\left(s\right)}\left(\Delta \nu ,P,T\right)+T_{M=+2}^{\left(s\right)}\left(\Delta \nu ,P,T\right) \end{array}$$

with $T_{M}^{\left(s\right)}$ calculated as described above (Equation (1)).

In order to determine isotope dependent relevant spectroscopic parameters between transitions, namely, frequency shift $\Delta \nu^{IS}=\nu^{o}{(}^{12}C)-\nu^{o}{(}^{13}C)$ and natural isotopic concentration ratio $R_{{}^{13}C{/}^{12}C}=a^{{}^{13}C}/a^{{}^{12}C}$ with $a^{IS}$ isotopic abundance of the IS isotope in the gas sample, the $T^{\left(s\right)}$ data are fitted to a function that describes the absorption modified FP Airy transmission at maximal optical resonance [29,35,45]:

(4) $$\begin{array}{ccc} T(\Delta \nu ,P,T) & = & K\sum_{M=0,+2}T_{M}\left(\Delta \nu ,P,T\right)+\left(A+B\Delta \nu \right) \end{array}$$

where a linear spectral background, with *A* and *B* as the frequency independent and slope parameters, respectively, and a scale factor *K* are considered to take into account possible not-compensated instrumental effects, due to the transmittance normalization. In Equation (4), $T_{M}$ is given by the following:

(5) $$\begin{array}{ccc} T_{M}\left(\Delta \nu ,P,T\right) & = & \frac{T_{\max}\left(\alpha_{M}\right)}{1+F\left(\alpha_{M}\right)\sin^{2}\left(\frac{\pi }{\Delta_{FSR}}\left(\Delta \nu \Delta n_{M}+\Delta_{lock}\right)\right)}\\ T_{\max}\left(\alpha_{M}\right) & = & \frac{T_{m}^{2}e^{-\alpha_{M}L}}{{\left(1-R_{m}e^{-\alpha_{M}L}\right)}^{2}}\\ F(\alpha_{M}) & = & 4R_{m}\frac{e^{-\alpha_{M}L}}{{\left(1-R_{m}e^{-\alpha_{M}L}\right)}^{2}} \end{array}$$

where $T_{m}$ and $R_{m}$ are the transmission and reflectivity coefficients of the FP mirrors, and $L=c/2\Delta_{FSR}$ is the cavity length. The argument of sin${}^{2}$ in the Airy function is the FP round trip phase shift, which is written as the sum of the empty cavity contribute, which is $\pi \Delta_{lock}/\Delta_{FSR}$ for the locked cavity, and $\pi \Delta \nu \Delta n_{M}/\Delta_{FSR}$, representing the contribution due to the gas dispersion for the *M* mode. $\Delta n_{M}$ and $\alpha_{M}$ are the dispersion and absorption coefficients, respectively, induced on the *M*-mode by resonant absorption transitions of the gas sample, which are calculated as a function of the laser detuning Δν and the thermodynamic conditions, *P* and *T*, from the real and imaginary part of FP’s refraction index variation: ${\left(n-1\right)}_{M}=\Delta n_{M}+i\alpha_{M}/2k_{M}$, with $k_{M}$ being the wave vector module of the absorbed *M* comb mode. Because $\Delta \nu^{IS}$ between the transitions is very large when compared to their linewidth in our thermodynamic conditions, any spectral interference effects between the two CO${}_{2}$ transitions can be safely neglected. Consequently, ${\left(n-1\right)}_{M}$ contributions for the M=0 and M=2 modes can be considered to be only induced separately by the ${}^{13}$CO${}_{2}$ and ${}^{12}$CO${}_{2}$ transitions, respectively, simplifying the analysis. If we label as t=a and t=b these ${}^{13}$CO${}_{2}$ and ${}^{12}$CO${}_{2}$ transitions, and considering a Voigt profile for the CO${}_{2}$ absorptions, we have the following:

(6) $$\begin{array}{ccc} \Delta n_{M}+i\frac{\alpha_{M}}{2k} & = & -\frac{cN\;a^{IS}\;S_{t}}{2\pi^{3/2}k_{M}\Delta \nu_{t}^{D}}\int_{-\infty }^{\infty }du\;\frac{e^{-u^{2}}}{\frac{\Delta \nu -\Delta \nu_{t}^{o}}{\Delta \nu_{t}^{D}}-u+i\frac{\Gamma_{t}/2}{\Delta \nu_{t}^{D}}} \end{array}$$

with the correspondence M=0,2⇔t=a,b. In Equation (6), *c* is the light speed, *N* is the numeric density of the gas at the given thermodynamic conditions, $a^{IS}$ is the abundance of the isotopologue IS in the gas mixture, S${}_{t}$ is the linestrength of the transition *t* per molecule [$S_{t}=S_{HI}/a^{IS}$, with $S_{HI}$ the linestrength value of the HITRAN database [44]. In this way, the isotopic abundances and their ratios can be measured independently of the transition’s levels], $\Delta \nu_{t}^{D}$ is the FWHM Doppler linewidth of the transition *t* of the isotopologue IS at *T*, $\Delta \nu_{t}^{o}$ is the transition frequency detuning, $\Gamma_{t}=\frac{\delta \Gamma_{t}}{\delta P}P$ is the FWHM collisional linewidth contribution at *P* and *T* and *u* represents the Doppler shift of each molecular class velocity.

The least-squared fit procedure of T (Equation (4)) to $T^{\left(s\right)}$ determines the best values of the molecular parameters, $\Delta \nu_{t}^{o}$, $S_{t}$, $a^{IS}$, $\Delta \nu_{t}^{D}$ and $\Gamma_{t}$ for both transitions as well as of the instrumental-related parameters $\Delta_{FSR}$, *F*, $\Delta_{lock}$, *K*, *A*, and *B*. As in previous measurements [29,35,45], two different fit strategies are performed. In the first approach, the final values of the relevant parameters are calculated from the weighted average of those values resulting from the fits of each individual scan at pressure *P*. In the other approach, all acquired spectra are considered in a single global fit, where some fit parameters (i.e., molecular-related parameters, $\Delta_{FSR}$, and *F*) are considered shared between all the scans, while $\Delta_{lock}$, *K*, *A*, and *B* are local parameters, considered to be different for each scan. In addition, parameters that can be evaluated independently, such as $\Delta_{FSR}$, $S_{t}$, and $\Delta \nu_{t}^{D}$ for both transitions are kept fixed during the fit procedure. The result of this global fit is graphically shown in Figure 3. A summary of the $\nu_{t}^{o}$, $a^{IS}$ and $\delta \Gamma_{t}/\delta P$ parameters for both transitions and the determined values $\Delta \nu^{IS}$ and $R_{{}^{13}C{/}^{12}C}$ from them are reported in Table 1. Their differences against the values reported in the HITRAN database [44] are also tabulated.

## 4. Discussion

Abundances of ${}^{12}$C${}^{16}$O${}_{2}$ and ${}^{13}$C${}^{16}$O${}_{2}$ are measured with a precision of 2.1% and 2.7%, respectively, in agreement with the HITRAN values. Consequently, a fractional isotope ratio $R_{{}^{13}C{/}^{12}C}$ = 0.0116 (4) is determined, as expected for a CO${}_{2}$ gas sample with the current natural isotopic content [44]. The present measurements can be considered a proof of principle of the DCVS applied to fractional isotopic ratio determinations, even if the experiment was performed at only one pressure, which was selected for giving the best precision performance for both isotopes in a single OFC scan. The final uncertainty is due to several issues: S/N ratio of each transmittance spectrum, limited scan of the full spectral profile of the transitions, and saturated absorption effects. The last two issues are particularly present in the ${}^{12}$C${}^{16}$O${}_{2}$ transition, which shows a quasi total saturation of the absorption, and the high frequency side is unrecorded. The final precision of $a^{{}^{12}C}$ determination is strongly limited by these experimental issues, which must be avoided to obtain the determinations for this isotope that are comparable to those achieved in other DFCDS [25]. Measurements at lower pressures could avoid saturation effects for this transition, paying for a decrease in the S/N ratio of the ${}^{13}$C${}^{16}$O${}_{2}$ transition by considering that the gas sample and absorption path are shared for both transitions. We are confident that measurements with gas sample pressure between 10 and 55 mbar could lead to a result that is compatible with the present uncertainties of the $a^{13^{C}}$. In addition, detection of the complete transition profiles would allow a significant improvement of the spectral parameters determination, including $a^{{}^{12}C}$, even at the gas pressure of the experiment. A more accurate choice of the frequency parameters of the OFC and FP could allow to center both transitions in the present maximum continuous scan of the OFC frequency for a single acquisition. Alternatively, consecutive OFC-shifted scans could be combined to increase the scan range. Through global fits, spectra recorded at different gas pressures, and involving other transitions of the sample gas, could be considered all together, increasing the final precision. Finally, combining detection and scan schemes as those described in [30,31] with the high Vernier filtering of our DCVS spectrometer, would allow faster broadband acquisitions.

Besides the fractional isotopic ratio, other spectral parameters of the targeted transitions are determined in our measurements. We find the transition frequencies to be in agreement with the HITRAN values, considering one standard deviation uncertainty. Their absolute value is reported with a precision of 1×10${}^{-8}$ and 4×10${}^{-8}$ for the ${}^{12}$C${}^{16}$O${}_{2}$ and ${}^{13}$C${}^{16}$O${}_{2}$ transitions, respectively. Instead, a small disagreement with the HITRAN values of the collisional broadening coefficients should be noted. Nevertheless, we believe that for our single pressure measurement, such discrepancies could be expected for a parameter that is just measuring a pressure-induced effect.

The present results show the capabilities of DCVS for precise measurements of the fractional isotopic ratios in a sample gas, with potential applicability in the detection of rare isotopologues [46], where absorption background from the others must be avoided. In principle, our technique could be applied to different kinds of OFC (ICL and QCL combs) in order to realize a compact setup toward the mid-infrared region.

## Figures and Tables

**Table 1:** $\Delta \nu^{IS}$ and $R_{{}^{13}C{/}^{12}C}$ determinations from the measured spectral parameters of the (20${}^{0}$1-00${}^{0}$0) R(18) of ${}^{13}$C${}^{16}$O${}_{2}$ and (20${}^{0}$1-00${}^{0}$0) P(45) of ${}^{12}$C${}^{16}$O${}_{2}$ transitions from DCVS measurements @ 5005 cm${}^{-1}$ and comparison with HITRAN database values [44]. Absolute frequencies are calculated by $\nu_{t}^{o}=N_{0}f_{r_{c}}+f_{o}+\Delta \nu_{t}^{o}$ with N${}_{0}$ order number of the OFC tooth transmitted by the M=0 FP mode and resonant with the ${}^{13}$C${}^{16}$O${}_{2}$ transition and f${}_{r_{c}}$ and $f_{o}$ repetition rate frequency at the center of the scan and offset frequency of the OFC, respectively. (Errors reported in parentheses). $\Delta_{\left(DCVS-HI\right)}$ are the differences between HITRAN database values and the present measured values for each tabulated parameter. The HITRAN database values are $\nu_{o}$ = 150041403.1 (2) MHz, $a^{{}^{13}C}$ = 0.01106 (1), δΓ/δP = 5.92 (5) MHz/mbar for the (20${}^{0}$1-00${}^{0}$0) R(18) transition of ${}^{13}$C${}^{16}$O${}_{2}$ and $\nu_{o}$ = 150,054,266.5 (2) MHz, $a^{{}^{12}C}$ = 0.9842 (9), δΓ/δP = 4.56 (4) MHz/mbar for the (20${}^{0}$1-00${}^{0}$0) P(45) transition of ${}^{12}$C${}^{16}$O${}_{2}$. The frequency shift between them is $\Delta \nu^{IS}$ = 12,863.5 (3) and the natural isotopic concentration ratio $R_{{}^{13}C{/}^{12}C}$ = 0.01123 (2). Errors of this differences take into account the error of the HITRAN values, which are added in quadrature to the measured ones.

Parameter	Global Fit	$\Delta_{\left(\mathrm{DCVS}-\mathrm{HI}\right)}$	Weighted-Average of Individual Fits	$\Delta_{\left(\mathrm{DCVS}-\mathrm{HI}\right)}$
$\Delta \nu^{IS}$ [MHz]	12,868.8 (6.4)	5.3 (6.4)	12,860.8 (6.0)	−2.7 (6.0)
$R_{{}^{13}C{/}^{12}C}$	0.0116(4)	0.0004 (4)	0.0116 (4)	0.0004 (4)
**Transition *a***	(20${}^{0}$1–00${}^{0}$0) R(18)	${}^{13}$C${}^{16}$O${}_{2}$		
$\nu^{o}$ [MHz]	150,041,400.3 (6.0)	−2.6 (6.0)	150,041,404.3 (5.6)	1.4 (5.6)
$a^{{}^{13}C}$	0.0109 (3)	−0.0002(3)	0.0107 (2)	−0.0004(2)
δΓ/δP [MHz/mbar]	5.3 (3)	−0.6 (3)	4.5 (3)	−1.4 (3)
**Transition *b***	(20${}^{0}$1–00${}^{0}$0) P(45)	${}^{12}$C${}^{16}$O${}_{2}$		
$\nu^{o}$ [MHz]	150,054,269.2 (2.0)	2.7 (2.0)	150,054,265.2 (2.1)	−1.3 (2.1)
$a^{{}^{12}C}$	0.94 (2)	−0.04(2)	0.92 (3)	−0.06 (3)
δΓ/δP [MHz/mbar]	4.0 (1)	−0.6 (1)	3.8 (2)	−0.8 (2)

## Data Availability

The data supporting this paper are available from the corresponding authors upon reasonable request.
